# Game-Based Learning Outcomes Among Physiotherapy Students: Comparative Study

**DOI:** 10.2196/26007

**Published:** 2021-03-24

**Authors:** Guadalupe Molina-Torres, Miguel Rodriguez-Arrastia, Raquel Alarcón, Nuria Sánchez-Labraca, María Sánchez-Joya, Pablo Roman, Mar Requena

**Affiliations:** 1 Department of Nursing, Physical Therapy and Medicine University of Almeria Almeria Spain; 2 Faculty of Health Sciences Pre-Department of Nursing Jaume I University Castellon de la Plana Spain; 3 Research Group CYS, Faculty of Health Sciences Jaume I University Castellon de la Plana Spain; 4 CTS-451, Health Sciences Research Group University of Almeria Almeria Spain; 5 Centro de Investigación en Salud (CEINSA), Health Sciences Centre Group University of Almeria Almeria Spain

**Keywords:** gamification, board game–based approach, health sciences, physiotherapy, teaching innovation

## Abstract

**Background:**

University teaching methods are changing, and in response to a classical teacher-centered approach, new methods continue to strengthen knowledge acquisition by involving students more actively in their learning, thus achieving greater motivation and commitment.

**Objective:**

This study aimed to analyze the degree of satisfaction of physiotherapy students who used a board game–based approach, as well as to compare the difference between traditional and gamification teaching methods and their influence on the final evaluation of these students.

**Methods:**

A comparative study was conducted. Participants were physiotherapy students who were enrolled in the subject of “physiotherapy in geriatric and adult psychomotricity” (n=59). They were divided into two groups (experimental [n=29] and control [n=30] groups) through convenience sampling. The experimental group received gamification lessons, where the students performed different tests adapted from Party&Co, and the control group received traditional lessons. A total of 16 theoretical lessons were received in both groups.

**Results:**

The scores in the final examination of the subject were higher in the experimental group (mean 7.53, SD 0.95) than in the control group (mean 6.24, SD 1.34), showing a statistically significant difference between the two groups (*P*=.001).

**Conclusions:**

Overall, the “Physiotherapy Party” game not only stimulated learning and motivated students, but also improved learning outcomes among participants, and the improvements were greater than those among students who received traditional teaching.

## Introduction

### Defining Concepts: Gamification Versus Game-Based Learning and Others

The term “gamification” emerged on the academic scene in the late 1990s, but was not commonly used in education and training until 2010 [1,2]. This concept describes an approach to teaching, in which students explore relevant aspects of games in a learning context designed by teachers and students in order to add depth and perspective to the experience of playing games. In this sense, this state-of-the-art teaching approach involves fun, engagement, significant learning, and interactive entertainment, and is defined as a type of gameplay that constitutes learning outcomes [2].

However, it is common to confuse different terms in these new trends, such as gamification, serious games, and game-based learning, which have sometimes been used interchangeably [1]. While gamification refers to the implementation of game mechanics in day-to-day processes or nongaming contexts, including the use of game components in different scenarios with no intention of creating a game [3,4], serious games are defined as games where the main goal is learning (not entertainment) and where designers do have the intention of creating a game [5]. Having said that, this may get slightly confusing because game-based learning also uses the aforementioned game-like elements and mechanics, but the differences are in gamification, which takes the entire learning process into a game [6]. Gamification typically involves the use of game mechanics or strategies (eg, rules and rewards), as well as visual and game-thinking elements (eg, cards and gameboards) to engage people and motivate and promote learning [7]. The most popular gamification tactics include (1) providing specific goals, (2) providing feedback, (3) showing progress, (4) providing badges of achievements, (5) using levels for incremental challenges, (6) giving a storyline, (7) allocating points, and (8) using a scoreboard [8].

In terms of making these elements more appealing, the rules are geared toward both processes and objectives, which might be fundamentally unplayable only by themselves. A recent systematic review of empirical evidence concluded that gamification has an impact on learning outcomes through motivation, academic achievement, and social connectivity. Gamification, used as part of a robust engagement strategy, is a motivator, both intrinsic and extrinsic, that plays a key role in promoting student engagement in learning. Additionally, there is a connection between learning achievement and engagement. The more engaged students are, the greater their achievement, and social comparison can explicitly promote social connectivity and a sense of relatedness among students [9].

Despite the promising data on gamification, there is limited experimental research and there are apparent limitations, such as lack of control groups, short interventions, and nonvalidated questionnaires [8]. According to a review by Arruzza et al [10], gamification appears to have several benefits for the general population, registered health professionals, undergraduate students in fields not related to health sciences, and undergraduate students in health sciences. Nevertheless, further research is needed to know if this results in increased levels of knowledge retention, application, and professional competence [10].

### Background

The lack of interaction between students and teachers, as well as between students themselves, is one of the most problematic scenarios nowadays [11,12]. In this context, where only student-content interaction exists, the frequency and intensity of educators’ influences on students using gamification are far greater [13]. In recent years, there has been a growing interest in how gamification can have a beneficial impact on student engagement in different educational settings [3,14,15], with a focus on learning through incentives [16]. In the current literature, however, there is scant evidence that connects gamification to traditional academic results or focuses on data from game-based initiatives as a source for education analytics [17]. Apart from that, it has been shown that the integration of game-based activities [18,19] does improve student enjoyment compared to traditional teaching approaches and provides more opportunities for class participation, hence increasing motivation and helping students learn more about the subject [20,21].

Consequently, university teaching approaches are adapting as educators aim to attain both better learning and more productive teaching [22]. State-of-the-art approaches, such as the use of gamification, seek to strengthen the acquisition of knowledge by engaging students in their own learning process as a response to a traditional approach, thereby gaining greater encouragement and confidence [23]. Thus, the aim of this study was to measure the learning experience of third-year physiotherapy students using the “Physiotherapy Party” game about geriatric and adult psychomotricity, which is a teaching game designed based on a gamification approach to assist them in their learning experience and help them prepare for their examination, as well as assess the results in the final evaluation of the subject. Our hypothesis was that the use of a game-based teaching approach will improve the motivation of students and involve them in their learning, even if it requires investing time to set up the game. Since the students who use the game will learn in a progressive and meaningful manner, the time and preparation will result in a higher grade in their final evaluation.

## Methods

### Study Design

**Figure 1:**
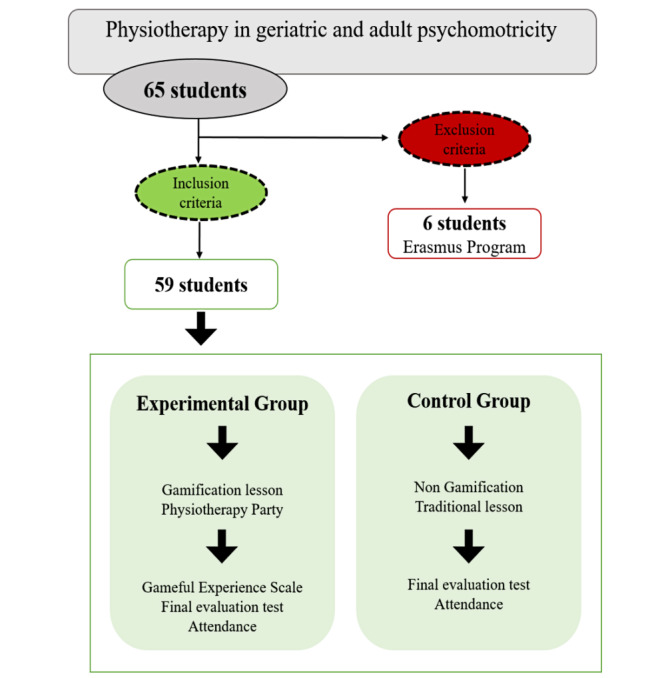
Flow diagram of participants.

A comparative design was used to study the experiences and results of a group of students. Of 65 students enrolled in the subject of “physiotherapy in geriatric and adult psychomotricity,” 59 were allocated into two different groups through convenience sampling. The groups were an experimental group (n=29) and a control group (n=30) (Figure 1). The experimental group (15 females and 14 males) received gamification lessons, and the control group (18 females and 12 males) received traditional lessons. A total of 16 theoretical lessons were received in both groups.

### Setting and Participants

The research population of this study consisted of physiotherapy students from the University of Almeria, and the study was carried out in the second semester of the 2018-19 academic year at the Faculty of Health Sciences. “Physiotherapy in geriatric and adult psychomotricity” is a compulsory subject of six European Credit Transfer and Accumulation System (ECTS) credits taught in the second semester of the third year, which has become essential for the aging population. This subject consists of theoretical and practical classes, which are divided into groups of 15 to 20 students. Its contents introduce students to the care of the elderly population; the physiology of aging and physiotherapy in traumatological and rheumatological conditions in the elderly population; neurological, cardiovascular, and respiratory diseases in the elderly population; cognitive and affective disorders in the elderly population; endocrine and nutritional disorders in the elderly population; urinary incontinence in the elderly population; and physical activity in the elderly population.

### Rules and Game Design of the Physiotherapy Students’ Party Game

**Figure 2:**
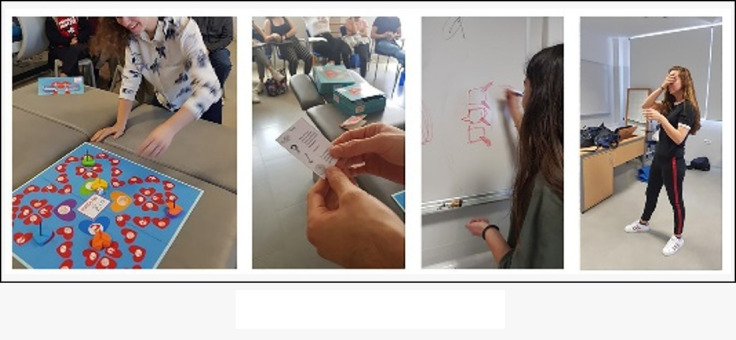
Physiotherapy Party game.

**Figure 3:**
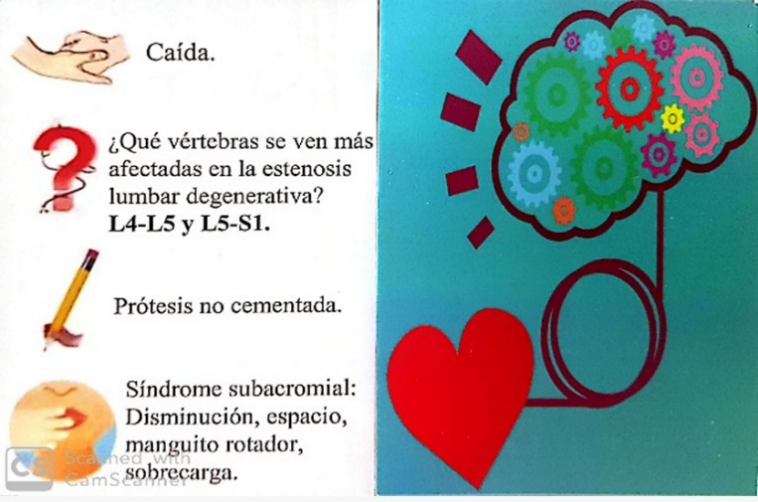
Card used in the game.

The “Physiotherapy Party” game was named “Guadaña&CO” (Figure 2). The aim of the game was for participants to win each one of the different challenges in mime, questions, forbidden words, and drawings to obtain cards in the main boxes and perform the final test. Students were divided into five groups to compete in the game (one group of five students and four groups of six students). These students had to show their knowledge in the subject to pass each of the four different tests on each card (Figure 3), which included the contents of the subject.

### Playing “Physiotherapy Party”

Once all elements of the game, such as the game board, tokens, dice, timer, card holder, and instruction sheet, were organized by two lecturers (GMT and PR), students were asked to create their own cards of each topic studied in class, which covered all the content of the subject. The purpose was to work and memorize basic concepts of the subject by making cards. Following the instructions of the “Physiotherapy Party” game, players first have to throw the dice and move their token through the game board. Then, they have to perform each of the tests that appear on the chosen cards from the corresponding box. The team has 30 seconds (controlled by an hour-glass timer) to carry out each one of the tests. Finally, the team that gets the last card out of each main box and performs the final test correctly wins.

### Variables and Data Collection

We collected sex and age as sociodemographic variables, as well as the attendance for gamification and traditional lessons, the grades in the final theoretical examination in the subject, and the game experience.

### Measures

The Gameful Experience (GAMEX) scale was used to assess the experience of physiotherapy students in the game [24,25]. This scale consists of 27 items graded on a Likert scale ranging from 1 (never) and 5 (always). The items are distributed into six different dimensions, which include enjoyment, absorption, creative thinking, activation, absence of negative effects, and dominance. The total Cronbach α value was .855. Particularly, the Cronbach α value was .843 for the enjoyment dimension, .898 for the absorption dimension, .865 for the creative thinking dimension, .790 for the activation dimension, .841 for the absence of negative effects dimension, and .860 for the dominance dimension [25].

### Final Evaluation

A 34-question test with four multiple-choice options on theoretical contents of the subject was developed for the final evaluation.

### Procedure

Before starting lessons with both groups, students were allocated on the basis of their subgroup enrolment. While subgroups 1 and 3 were assigned to the experimental group, subgroups 2 and 4 were assigned to the control group and were not informed of the “Physiotherapy Party” game. The difference between the two groups was that the experimental group used the “Physiotherapy Party” game, whereas the control group did not use this game. There were no further differences (evaluation, lessons, contents, lecturer, etc). Students were informed about the aim of the investigation, as well as the confidential and anonymous treatment of their data. Once informed consent was obtained, the gamification lessons and traditional lessons were started. The study data were collected in June 2019, after the last gamification and traditional lesson, as well as after the final theoretical examination.

### Data Analysis

The data analysis was carried out using the statistical software SPSS version 22 (IBM Corp). First, a descriptive analysis was conducted from the results. Central tendency and dispersion measures were determined for the quantitative variables, while the frequency and percentage were analyzed for the categorical variables. In contrasting the hypothesis between qualitative and quantitative variables, after showing a normal distribution with the Kolmogorov-Smirnov test, the Student *t* parametric test was used for independent samples.

### Ethical Considerations

This study was approved by the Ethics Committee of the University of Almeria (Spain) (EFM-28/19). All participants were informed about the aim of the study prior to participation. Participants were informed about the confidentiality of their data, and all consent forms were signed. All ethical aspects established in the Declaration of Helsinki were followed at all times.

## Results

### Sociodemographic Characteristics

Fifty-nine physiotherapy students from the University of Almeria participated in this study. Table 1 shows the sociodemographic characteristics of the participants. The total sample consisted of 59 students (55.9% [n=33] were female and 44.1% [n=26] were male), with a mean age of 23.37 years (SD 4.91 years).

**Table 1:** Sociodemographic characteristics of the participants (N=59).

Characteristic		Experimental group	Control group
Participants, n (%)		29 (49.2%)	30 (50.8%)
**Sex, n (%)**			
	Female	15 (51.7%)	18 (60.0%)
	Male	14 (48.3%)	12 (40.0%)
Age (years), mean (SD)		22.10 (3.39)	24.60 (5.83)

### Attendance for Gamification and Traditional Lessons

Out of 16 theory and gamification sessions, the mean attendance by students in both groups was 10.85 lessons (SD 4.84), while that in the experimental group was 12.66 (SD 3.06) and that in the control group was 9.10 (SD 5.60). Both groups showed a significant difference in relation to their attendance for the traditional lesson (*t*_57_=3.009, *P*=.004). 

### Results in the Final Qualification

Regarding the final qualification of the theoretical examination, the final mean score was 6.87 (SD 1.32) for both groups, while that in the experimental group was 7.53 (SD 0.95) and that in the control group was 6.24 (SD 1.34). Final scores in both groups showed significant differences (gamification was either applied or not applied) (*t*_57_=4.208, *P*=.001).

### GAMEX Scale

Considering the GAMEX scale (Table 2), the scores were above average in all the dimensions, except negative effects. Specifically, the mean scores were as follows: enjoyment, 25.93 (SD 4.01; range 6-30); absorption, 20.03 (SD 5.87; range 6-30); creative thinking, 14.10 (SD 3.42; range 4-20); activation, 15.10 (SD 2.89; range 4-20); absence of negative effects, 5.14 (SD 2.23; range 3-15); and dominance, 12.79 (SD 3.66; range 4-20).

**Table 2:** Data of each Gameful Experience (GAMEX) scale dimension by sex.

Dimension	Score, mean (SD)		*P* value
	Men	Women	
Enjoyment	26.00 (3.94)	25.87 (4.22)	.93
Absorption	19.43 (5.47)	20.60 (6.35)	.60
Creative thinking	14.57 (3.22)	13.67 (3.65)	.49
Activation	15.00 (2.74)	15.20 (3.12)	.86
Absence of negative effects	4.79 (2.75)	5.47 (1.64)	.42
Dominance	13.64 (3.05)	12.00 (4.10)	.24

## Discussion

### Principal Findings

The aim of this study was to analyze the degree of satisfaction of students after using the “Physiotherapy Party” game in geriatric and adult psychomotricity, as well as to compare the two different teaching methods (traditional lessons and gamification lessons) and their impacts on the final evaluation.

To the best of our knowledge, there is no previous evidence on the use of this classic family board game adapted for teaching in physiotherapy. This innovative educational game is trendy, enjoyable, and entertaining as the first dimension of the GAMEX scale showed. As our participants reported in the satisfaction questionnaire, it enabled them to remember and apply knowledge more easily. In the same line of results, previous reports on the use of gamification in nursing studies support these findings, such as using an escape room among nursing students [22,26], in which gamification helped to acquire competencies by positively shaping the teaching-learning process. Gamification has been shown to successfully increase students’ motivation to learn and to be an additional resource based on student engagement and meaningful learning in education [27]. In this sense, Cain [28] pointed out that it can be used to have a more positive student experience as it immerses students in their learning process as active participants. Among medical students, Kinio et al [29] developed a platform for learning that may be more enjoyable and serve as a complement to traditional lectures. These platforms might be used to do quick assessments in learning activities, such as the game “Kahoot!” [30], which have led to a paradigm change in classrooms, promoting self-learning among students.

In addition, these kinds of games promote creative thinking, as reflected in the results of this study and those of other studies [31-34]. In line with the findings of Gómez-Urquiza et al [22], games, such as Escape Room and the one suggested in this study, help students in their day-to-day practice when it comes to thinking critically, as they have to bear in mind all potential solutions to their problems, which enhances their overall decision-making skills [35]. Additionally, this gamification-like approach promotes the application of knowledge acquired in the course of using the platform [22].

Analyzing other dimensions of the gaming experience, the participants did not feel frustrated in most situations. These results are similar to those found by other studies [36], where only a few students showed frustration and confusion.

Regarding the learning results, there was a significant difference between both groups, and the group involving gamification had the best results. In line with these results, other studies have shown the benefits of such game-based learning approaches over traditional strategies [37]. Game-based strategies have been shown to encourage learning and thus result in dramatically improved student performance [38]. In the end, game-based teaching approaches enhance student motivation while increasing student participation and providing effective feedback [39]. These interventions have been shown to have a beneficial impact across educational programs for learners, with improved grade point averages [40], increased positive student perception of learning [41], better understanding of concepts [42], and decreased course drop-out rates [43] compared to the findings among students who used more traditional approaches.

### Methodological Considerations

There are several limitations to be taken into account in the results of this study. First, the generalization of our results should be considered cautiously, as participation in the study was voluntary and sample selection was through convenience sampling. Furthermore, the sample was not randomized and only 59 students participated. Additionally, owing to the nature of the intervention, the participants could not be blinded. Besides, the GAMEX scale has been validated in a digital environment and has not been validated for a nondigital game, although it was previously used with good consistency [44,45]. Despite these drawbacks, the limited number of studies exploring the influence of games in the health sciences is one of the strengths. In this sense, there is no evidence of the use of gamification in physiotherapy, and therefore, it offers an opportunity as a new research line in innovative teaching. Moreover, it should be emphasized that the use of the “Physiotherapy Party” game improves academic performance with respect to traditional education. However, this innovative teaching approach initially requires a considerable amount of time for the teacher to prepare game materials, rules, and game dynamics. Students must also prepare game cards based on the content of the subject, although they are indeed working and studying, which promotes a gradual and meaningful learning process. Conversely, enjoyment was not measured at each session. It would therefore be interesting to measure this dimension at each session in order to assess the appropriate number of sessions for the use of this innovative teaching method.

### Conclusions

The “Physiotherapy Party” game was shown to be a learning activity that allows students to remember and implement the knowledge and professional competences acquired in the subject more easily. In addition, it motivates students to study, thus improving their attendance in these classes.

This study confirms that gamification as an alternative to conventional approaches can be considered an interesting and state-of-the-art approach for teaching physiotherapy, which can be used at the same time to improve knowledge among university students.

## Abbreviations

GAMEX: Gameful Experience
